# Supplementary material for: Molecular basis of positional memory in limb regeneration
Source: Nature. 2025 May 21;642(8068):730–8. doi: 10.1038/s41586-025-09036-5 (PMC12176643; doi:10.1038/s41586-025-09036-5)
Supplement: Supplementary file 1 — Gating strategy for the flow cytometry of Hand2:EGFP cells. a, Representative plots depicting gates defined for all cells (left), single cells (centre) and GFP-positive cells (right). Depicted are plots from the 14 d.p.a. blastema sample, which harbours Hand2:EGFP+ cells. b, Representative plots depicting gates defined for all cells (left), single cells (centre) and GFP+ cells (right). Depicted are plots from the 0 d.p.a. negative control sample, which does not harbour Hand2:EGFP+ cells. Plots were assembled in FLOWJO (BD Biosciences) [file 41586_2025_9036_MOESM1_ESM.pdf]

nature portfolio

https://doi.org/10.1038/s41586-025-09036-5

Supplementary information

Molecular basis of positional memory in limb regeneration

In the format provided by the authors and unedited

Supplementary Fig. 1 | Gating strategy for flow cytometry of *Hand2*:EGFP cells.

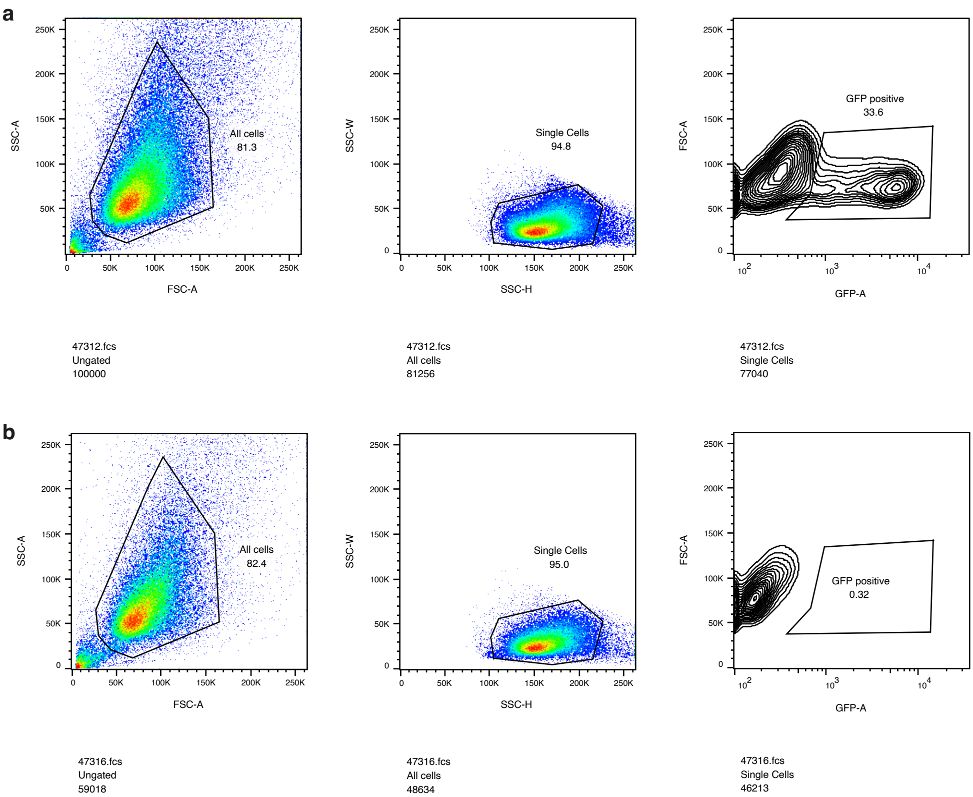

Supplementary Fig. 1 | Gating strategy for flow cytometry of *Hand2*:EGFP cells.
a, Representative plots depicting gates defined for all cells (left), single cells (centre) and GFP-positive cells (right). Depicted are plots from the 14 dpa blastema sample, which harbour *Hand2*:EGFP+ cells. **b,** Representative plots depicting gates defined for all cells (left), single cells (centre) and GFP-positive cells (right). Depicted are plots from the 0 dpa negative control sample, which does not harbour *Hand2*:EGFP+ cells. Plots were assembled in FLOWJO (BD Biosciences).
