# Supplementary material for: Molecular basis of positional memory in limb regeneration
Source: Nature. 2025 May 21;642(8068):730–8. doi: 10.1038/s41586-025-09036-5 (PMC12176643; doi:10.1038/s41586-025-09036-5)
Supplement: Supplementary file 2 — Reporting Summary [file 41586_2025_9036_MOESM2_ESM.pdf]

nature portfolio

Corresponding author(s): Elly M Tanaka, Leo Otsuki

Last updated by author(s): Mar 12, 2025

Reporting Summary

Nature Portfolio wishes to improve the reproducibility of the work that we publish. This form provides structure for consistency and transparency in reporting. For further information on Nature Portfolio policies, see our Editorial Policies and the Editorial Policy Checklist.

Statistics

For all statistical analyses, confirm that the following items are present in the figure legend, table legend, main text, or Methods section.

n/a	Confirmed	
☐	☒	The exact sample size (*n*) for each experimental group/condition, given as a discrete number and unit of measurement
☐	☒	A statement on whether measurements were taken from distinct samples or whether the same sample was measured repeatedly
☐	☒	The statistical test(s) used AND whether they are one- or two-sided<br>*Only common tests should be described solely by name; describe more complex techniques in the Methods section.*
☐	☒	A description of all covariates tested
☐	☒	A description of any assumptions or corrections, such as tests of normality and adjustment for multiple comparisons
☐	☒	A full description of the statistical parameters including central tendency (e.g. means) or other basic estimates (e.g. regression coefficient) AND variation (e.g. standard deviation) or associated estimates of uncertainty (e.g. confidence intervals)
☐	☒	For null hypothesis testing, the test statistic (e.g. *F*, *t*, *r*) with confidence intervals, effect sizes, degrees of freedom and *P* value noted<br>*Give P values as exact values whenever suitable.*
☐	☒	For Bayesian analysis, information on the choice of priors and Markov chain Monte Carlo settings
☐	☒	For hierarchical and complex designs, identification of the appropriate level for tests and full reporting of outcomes
☒	☐	Estimates of effect sizes (e.g. Cohen's *d*, Pearson's *r*), indicating how they were calculated

Our web collection on statistics for biologists contains articles on many of the points above.

Software and code

Policy information about availability of computer code

Data collection	No custom software was used for data collection.
Data analysis	Differential expression analysis of RNA-sequencing data.<br>Adaptor sequences were trimmed from the raw sequencing reads with Trimmomatic (version 0.39), using parameters: ILLUMINACLIP:Adapters.fa:2:30:7 SLIDINGWINDOW:4:20 MINLEN:40 in single-end mode. Trimmed sequenced reads were mapped to axolotl genome AmexG_v6.0-DD with HISAT2, using parameters: --no-unal-summary-file Output.log -k 5-very-sensitive -x DBGenome -U Reads.fq.gz Alignment.sam. featureCounts was used to generate a read counts table. Differential expression analysis was performed on 2 anterior replicates and 3 posterior replicates using R version 4.1.2 and DESeq2 version 1.34.0 with an FDR cutoff of p < 0.01. Volcano plots were generated using ggplot2 version 3.3.6. Heatmaps were generated with the pheatmap package version 1.0.12 (R. Kolde). Gene Ontology analysis was performed with the topGO package version 2.46.0 (Alexa A, Rahnenfuhrer J) using parameters: ontology= "BP", geneSelectionFun = topDiffGenes, an not= annFUN.org, mapping= "org.Hs.eg.db". To calculate significant GO terms, Fisher's exact test was performed with the "elim" algorithm. To facilitate interpretation of the differential expression results, we generated a custom gene nomenclature derived from the AmexT _ v47 transcriptome. We concatenated each axolotl gene identifier with the gene symbol for the direct human homologue where available or, if not available, the closest homologue from the NCBI non-redundant database.<br><br>Determination of axolotl ZRS enhancer<br>The axolotl ZRS enhancer was determined by multiple species alignment of the following genome sequences using mVISTA (https://genome.lbl.gov/vista/mvista/submit.shtml) and PipMaker (http://pipmaker.bx.psu.edu/pipmaker/).<br>Axolotl (Ambystoma mexicanum) assembly AmexG_v6.0-DD chr2p:694366863-694689506<br>Human (Homo sapiens) assembly hg38 chr7:156769228-156790956<br>Mouse (Mus musculus) assembly mm10 chr5:29292950-29323801

Chick (Gallus gallus) assembly Gal6 chr2:8538956-8559114
Fugu (Takifugu rubripes) assembly fr3 chr10:5739579-5747090

Estimation of indel frequency in Hand2 CRISPants
Indel frequency was estimated from Sanger sequencing results using the ICE Analysis Tool (Synthego, https://ice.synthego.com/).

Image analysis
Microscope images were analysed using ZEN software (Zeiss) or Fiji software 2.14.0/1.54f.

Statistical analysis and data representation
Statistical analyses and graph plotting was performed using Prism software v10 (GraphPad). Data were tested for assumptions of normality and equality of variance to determine the appropriate statistical tests to perform. No data were excluded. Mean values are reported +/- standard deviation (SD). Statistical significance was defined as p < 0.05. All figures were assembled in Adobe Illustrator 2023.

For manuscripts utilizing custom algorithms or software that are central to the research but not yet described in published literature, software must be made available to editors and reviewers. We strongly encourage code deposition in a community repository (e.g. GitHub). See the Nature Portfolio guidelines for submitting code & software for further information.

Data

Policy information about availability of data

All manuscripts must include a data availability statement. This statement should provide the following information, where applicable:

- Accession codes, unique identifiers, or web links for publicly available datasets
- A description of any restrictions on data availability
- For clinical datasets or third party data, please ensure that the statement adheres to our policy

Genome assembly AmexG_v6.0-DD (https://genome.axolotl-omics.org/index.html) and transcriptome assembly AmexT_v47 (https://www.axolotl-omics.org/assemblies) were used (Schloissnig et al. 2021).

All RNA-sequencing data have been deposited at the Gene Expression Omnibus (GEO), under accessions GSE243137 (dermal cell data) and GSE284768 (all other data).

There are no restrictions on data availability.

Research involving human participants, their data, or biological material

Policy information about studies with human participants or human data. See also policy information about sex, gender (identity/presentation), and sexual orientation and race, ethnicity and racism.

Reporting on sex and gender	Not applicable to this study as no human data were collected.
Reporting on race, ethnicity, or other socially relevant groupings	Not applicable to this study as no human data were collected.
Population characteristics	Not applicable to this study as no human data were collected.
Recruitment	Not applicable to this study as no human data were collected.
Ethics oversight	Not applicable to this study as no human data were collected.

Note that full information on the approval of the study protocol must also be provided in the manuscript.

Field-specific reporting

Please select the one below that is the best fit for your research. If you are not sure, read the appropriate sections before making your selection.

☒ Life sciences ☐ Behavioural & social sciences ☐ Ecological, evolutionary & environmental sciences

For a reference copy of the document with all sections, see nature.com/documents/nr-reporting-summary-flat.pdf

Life sciences study design

All studies must disclose on these points even when the disclosure is negative.

Sample size	No sample size calculation was performed. All sample sizes are indicated in the relevant figure legend, main text or methods. n numbers refer to independent biological replicates. A minimum of 3 biological replicates were used per condition, which we considered a balance between data reliability and animal usage.
Data exclusions	No data were excluded.

Replication	Each experiment was replicated at least once unless indicated otherwise, with successful replication of conclusions. Conclusions were made based on experiments using independent axolotl cohorts and multiple experimental approaches.
Randomization	Axolotls from the same cohort were randomly allocated into experimental or control groups and housed under identical conditions. Exceptions are experiments (detailed in the Methods) in which the control was performed on one limb and the experimental perturbation on the other limb of the same axolotl. For ALM experiments, we ensured that there was no bias in the success of ALM formation based on left or right host limb.
Blinding	Blinding was not feasible as the work was largely performed by one author and/or phenotypes were much more severe in the experimental cohort than in the control cohort.

Reporting for specific materials, systems and methods

We require information from authors about some types of materials, experimental systems and methods used in many studies. Here, indicate whether each material, system or method listed is relevant to your study. If you are not sure if a list item applies to your research, read the appropriate section before selecting a response.

Materials & experimental systems

n/a	Involved in the study
☐	☒ Antibodies
☒	☐ Eukaryotic cell lines
☒	☐ Palaeontology and archaeology
☐	☒ Animals and other organisms
☒	☐ Clinical data
☒	☐ Dual use research of concern
☒	☐ Plants

Methods

n/a	Involved in the study
☒	☐ ChIP-seq
☐	☒ Flow cytometry
☒	☐ MRI-based neuroimaging

Antibodies

Antibodies used	Rabbit anti-Prrx1 antibody, generated by Gerber et al. 2018 (DOI: 10.1126/science.aaq0681). Mouse anti-Col1A1 antibody (SP1.D8, DSHB). Alexa 647-conjugated anti-rabbit (Invitrogen A-21244) or anti-mouse (Invitrogen A-21240) secondary antibody.
Validation	Prrx1 antibody was characterised in axolotl limb tissue by Gerber et al. 2018 (DOI: 10.1126/science.aaq0681). Col1A1 antibody was characterised in axolotl tissue by Gerber et al. 2018 (DOI: 10.1126/science.aaq0681).

Animals and other research organisms

Policy information about studies involving animals; ARRIVE guidelines recommended for reporting animal research, and Sex and Gender in Research

Laboratory animals	Axolotl (Ambystoma mexicanum), age up to ~8 months.
Wild animals	The study did not involve wild animals.
Reporting on sex	Sex data was not collected in this study. Sex is not readily distinguishable based on morphology in the axolotl sizes used in this study. Thus, data collection was randomised with respect to sex. Sex determination in these animals requires a genotyping PCR, and the injury from tissue harvesting could induce an unwanted tissue response, as suggested in DOI: 10.1016/j.ydbio.2017.07.010.
Field-collected samples	The study did not involve animals collected from the field.
Ethics oversight	All animal experiments were approved by the Magistrate of Vienna (Genetically Modified Organism Office and MA58, City of Vienna, Austria), under licenses GZ:51072/2019/16, GZ: MA58-1432587-2022-12 and GZ: MA58-1516101-2023-21.

Note that full information on the approval of the study protocol must also be provided in the manuscript.

Plants

Seed stocks	Not applicable.
Novel plant genotypes	Not applicable.
Authentication	Not applicable.

Flow Cytometry

Plots

Confirm that:

- [x] The axis labels state the marker and fluorochrome used (e.g. CD4-FITC).
- [x] The axis scales are clearly visible. Include numbers along axes only for bottom left plot of group (a 'group' is an analysis of identical markers).
- [x] All plots are contour plots with outliers or pseudocolor plots.
- [x] A numerical value for number of cells or percentage (with statistics) is provided.

Methodology

Sample preparation	The Hand2:EGFP intensity of mature arm cells, 7 dpa blastema cells and 14 dpa blastema cells were compared by flow cytometry. Lower arm tissue was harvested from 6 cm Hand2:EGFP axolotls. The entire lower arm was taken for mature measurements. Blastemas were generated by amputating through the middle of the lower arm 7 or 14 days prior to flow cytometry. Harvested tissues were dissociated into single cell suspensions using Liberase TM enzyme (Merck 05401127001) as described in Lin et al. 2021 (DOI: 10.1016/j.devcel.2021.04.016), with the following modifications: dissociation was performed for 55 mins (mature sample) or 45 mins (blastema samples) and the cells were filtered through a 70 um MACS SmartStrainer (Miltenyi Biotec 130-098-462).
Instrument	Cells were analysed by FACS (FACSAria III Cell Sorter, BD Biosciences) using a 100 um low pressure nozzle.
Software	FLOWJO software (BD Biosciences).
Cell population abundance	Representative population abundances are depicted in Supplementary Fig. 1. Population abundance generally ranged from ~5-70% depending on genotype and experiment. Purity was not assessed by re-sorting post-sort cells.
Gating strategy	The gating strategy is depicted in Supplementary Fig. 1. Cells were identified based on FSC-A and SSC-A gates, and doublet exclusion performed using SSC-H and SSC-W gates. Cell purity was generally > 80% and the proportion of single cells post-dissociation > 90%. The population of interest was identified by comparing against a parallel treated negative control sample lacking the relevant fluorescent protein, as indicated in the manuscript. This allowed determination of positive and negative cells.

- [x] Tick this box to confirm that a figure exemplifying the gating strategy is provided in the Supplementary Information.
