# Supplementary material for: Molecular basis of positional memory in limb regeneration
Source: Nature. 2025 May 21;642(8068):730–8. doi: 10.1038/s41586-025-09036-5 (PMC12176643; doi:10.1038/s41586-025-09036-5)
Supplement: Supplementary file 4 — Supplementary Tables 1–18 [file 41586_2025_9036_MOESM4_ESM.zip › 2023-09-17078B-s4/SupplementaryTableLegends.docx]

**Supplementary Information**

**Molecular basis of positional memory in limb regeneration**

**Otsuki, L *^1,2,*^*, Plattner, SA *^1,2^*, Taniguchi-Sugiura, Y *^1,2^*, Falcon, F *^1,2^*, Tanaka, EM *^1,2,^*^*^**

^1^ Institute of Molecular Biotechnology of the Austrian Academy of Sciences (IMBA), Vienna BioCenter (VBC), Dr. Bohr-Gasse 3, 1030 Vienna, Austria

^2^ Research Institute of Molecular Pathology (IMP), Campus-Vienna-Biocenter 1, 1030 Vienna, Austria

^+^ Current address: Institute of Molecular Biotechnology of the Austrian Academy of Sciences (IMBA), Vienna BioCenter (VBC), Dr. Bohr-Gasse 3, 1030 Vienna, Austria

^*^ Authors for correspondence

Legends for Supplementary Tables 1-18 Page 2

Legend for Supplementary Figure 1 Page 4

**Legends for Supplementary Tables 1-18**

**Supplementary Table 1 | Differentially expressed genes enriched in posterior dermal cells, ordered by p-value.**

Table of differentially expressed genes enriched in posterior dermal cells compared to anterior dermal cells. Genes are ordered by decreasing statistical significance (top to bottom). Differential expression was determined statistically using DESeq2 (two-tailed Wald test with Benjamini-Hochberg adjustment for multiple testing), with a FDR cutoff of *p*<0.01.

**Supplementary Table 2 | Differentially expressed genes enriched in anterior dermal cells, ordered by p-value.**

Table of differentially expressed genes enriched in anterior dermal cells compared to posterior dermal cells. Genes are ordered by decreasing statistical significance (top to bottom). Differential expression was determined statistically using DESeq2 (two-tailed Wald test with Benjamini-Hochberg adjustment for multiple testing), with a FDR cutoff of *p*<0.01.

**Supplementary Table 3 | Differentially expressed genes belonging to the GO term category “Extracellular matrix”.**

Table of differentially expressed genes belonging to the GO term category “Extracellular matrix”. Differential expression was determined statistically using DESeq2 (two-tailed Wald test with Benjamini-Hochberg adjustment for multiple testing), with a FDR cutoff of *p*<0.01. Positive fold change values indicate genes enriched in posterior dermal cells compared to anterior dermal cells.

**Supplementary Table 4 | Differentially expressed genes belonging to the GO term category “Cell adhesion”.**

Table of differentially expressed genes belonging to the GO term category “Cell adhesion”. Differential expression was determined statistically using DESeq2 (two-tailed Wald test with Benjamini-Hochberg adjustment for multiple testing), with a FDR cutoff of *p*<0.01. Positive fold change values indicate genes enriched in posterior dermal cells compared to anterior dermal cells.

**Supplementary Table 5 | Expression of spatial transcription factor genes in *Hand2*-misexpressing cells compared to mCherry controls.**

Expression of anterior and posterior transcription factors in *Prrx1*>mCherry-*Hand2* cells versus *Prrx1*>mCherry controls. A positive log2FoldChange indicates higher expression in *Hand2*-misexpressing cells. Differential expression was determined statistically using DESeq2 (two-tailed Wald test with Benjamini-Hochberg adjustment for multiple testing), with a *padj* < 0.05. Significant genes are bolded.

**Supplementary Table 6 | Expression of spatial transcription factor genes in *Hand2*-misexpressing cells compared to anterior blastema cells.**

Expression of anterior and posterior transcription factors in *Prrx1*>mCherry-*Hand2* cells versus anterior ‘A’ blastema cells isolated from double transgenic axolotls. A positive log2FoldChange indicates higher expression in *Hand2*-misexpressing cells. Differential expression was determined statistically using DESeq2 (two-tailed Wald test with Benjamini-Hochberg adjustment for multiple testing), with a *padj* < 0.05. Significant genes are bolded.

**Supplementary Table 7 | All differentially expressed genes upregulated in *Hand2*-misexpressing cells compared to mCherry controls.**

A list of all genes upregulated in *Prrx1*>mCherry-*Hand2* cells compared to *Prrx1*>mCherry controls. A positive log2FoldChange indicates higher expression in *Hand2*-misexpressing cells. Differential expression was determined statistically using DESeq2 (two-tailed Wald test with Benjamini-Hochberg adjustment for multiple testing), with a *padj* < 0.05.

**Supplementary Table 8 | All differentially expressed genes downregulated in *Hand2*-misexpressing cells compared to mCherry controls.**

A list of all genes downregulated in *Prrx1*>mCherry-*Hand2* cells compared to *Prrx1*>mCherry controls. A negative log2FoldChange indicates higher expression in mCherry control cells. Differential expression was determined statistically using DESeq2 (two-tailed Wald test with Benjamini-Hochberg adjustment for multiple testing), with a *padj* < 0.05.

**Supplementary Table 9 | All differentially expressed genes upregulated in *Hand2*-misexpressing cells compared to anterior blastema cells.**

A list of all genes upregulated in *Prrx1*>mCherry-*Hand2* cells compared to anterior ‘A’ blastema cells isolated from double transgenic axolotls. A positive log2FoldChange indicates higher expression in *Hand2*-misexpressing cells. Differential expression was determined statistically using DESeq2 (two-tailed Wald test with Benjamini-Hochberg adjustment for multiple testing), with a *padj* < 0.05.

**Supplementary Table 10 | All differentially expressed genes downregulated in *Hand2*-misexpressing cells compared to anterior blastema cells.**

A list of all genes downregulated in *Prrx1*>mCherry-*Hand2* cells compared to anterior ‘A’ blastema cells isolated from double transgenic axolotls. A negative log2FoldChange indicates higher expression in anterior blastema cells. Differential expression was determined statistically using DESeq2 (two-tailed Wald test with Benjamini-Hochberg adjustment for multiple testing), with a *padj* < 0.05.

**Supplementary Table 11 | Loadings of genes contributing to principal component 2 (PC2) in the cell transplantation PCA.**

The top 1000 most variable genes were used as an input. Genes are ordered by their contribution to PC2. PC1 is also indicated.

**Supplementary Table 12 | Expression of spatial transcription factor genes in A->P transplanted cells compared to anterior blastema cells.**

Expression of anterior and posterior transcription factors in A->P transplanted cells versus anterior ‘A’ blastema cells isolated from double transgenic axolotls. A positive log2FoldChange indicates higher expression in A->P cells. Differential expression was determined statistically using DESeq2 (two-tailed Wald test with Benjamini-Hochberg adjustment for multiple testing), with a *padj* < 0.05. Significant genes are bolded.

**Supplementary Table 13 | Anterior or posterior blastema-specific genes that are differentially expressed in A->P transplanted cells.**

Anterior and posterior blastema-specific genes were identified by DESeq2 (two-tailed Wald test with Benjamini-Hochberg adjustment for multiple testing) between A and P cell samples with a *padj*<0.05. These genes were overlapped with genes that were up- or down- regulated in A>P transplanted cells compared to A cell controls.

**Supplementary Table 14 | Expression of spatial transcription factor genes in A->P transplanted cells compared to A->A transplanted cells.**

Expression of anterior and posterior transcription factors in A->P transplanted cells versus A->A transplanted cells. A positive log2FoldChange indicates higher expression in A->P cells. Differential expression was determined statistically using DESeq2 (two-tailed Wald test with Benjamini-Hochberg adjustment for multiple testing), with a *padj* < 0.05. Significant genes are bolded.

**Supplementary Table 15 | All differentially expressed genes upregulated in A->P transplanted cells compared to A->A controls.**

A list of all genes upregulated in A->P transplanted cells compared to A->A control cells. A positive log2FoldChange indicates higher expression in A->P cells. Differential expression was determined statistically using DESeq2 (two-tailed Wald test with Benjamini-Hochberg adjustment for multiple testing), with a *padj* < 0.05.

**Supplementary Table 16 | All differentially expressed genes downregulated in A->P transplanted cells compared to A->A controls.**

A list of all genes downregulated in A->P transplanted cells compared to A->A control cells. A negative log2FoldChange indicates higher expression in A->A cells. Differential expression was determined statistically using DESeq2 (two-tailed Wald test with Benjamini-Hochberg adjustment for multiple testing), with a *padj* < 0.05.

**Supplementary Table 17 | All differentially expressed genes upregulated in A->P transplanted cells compared to anterior blastema cells.**

A list of all genes upregulated in A->P transplanted cells compared to anterior ‘A’ blastema cells isolated from double transgenic axolotls. A positive log2FoldChange indicates higher expression in A->P cells. Differential expression was determined statistically using DESeq2 (two-tailed Wald test with Benjamini-Hochberg adjustment for multiple testing), with a *padj* < 0.05.

**Supplementary Table 18 | All differentially expressed genes downregulated in A->P transplanted cells compared to anterior blastema cells.**

A list of all genes downregulated in A->P transplanted cells compared to anterior ‘A’ blastema cells isolated from double transgenic axolotls. A negative log2FoldChange indicates higher expression in anterior blastema cells. Differential expression was determined statistically using DESeq2 (two-tailed Wald test with Benjamini-Hochberg adjustment for multiple testing), with a *padj* < 0.05.

**Legend for Supplementary Figure 1**

**Supplementary Fig. 1 | Gating strategy for flow cytometry of *Hand2*:EGFP cells.
a,** Representative plots depicting gates defined for all cells (left), single cells (centre) and GFP-positive cells (right). Depicted are plots from the 14 dpa blastema sample, which harbour *Hand2*:EGFP+ cells. **b,** Representative plots depicting gates defined for all cells (left), single cells (centre) and GFP-positive cells (right). Depicted are plots from the 0 dpa negative control sample, which does not harbour *Hand2*:EGFP+ cells. Plots were assembled in FLOWJO (BD Biosciences).
